# Overt Attention and Context Factors: The Impact of Repeated Presentations, Image Type, and Individual Motivation

**DOI:** 10.1371/journal.pone.0021719

**Published:** 2011-07-05

**Authors:** Kai Kaspar, Peter König

**Affiliations:** Institute of Cognitive Science, University of Osnabrück, Osnabrück, Germany; Georgia Health Sciences University, United States of America

## Abstract

The present study investigated the dynamic of the attention focus during observation of different categories of complex scenes and simultaneous consideration of individuals' memory and motivational state. We repeatedly presented four types of complex visual scenes in a pseudo-randomized order and recorded eye movements. Subjects were divided into groups according to their motivational disposition in terms of action orientation and individual rating of scene interest.

Statistical analysis of eye-tracking data revealed that the attention focus successively became locally expressed by increasing fixation duration; decreasing saccade length, saccade frequency, and single subject's fixation distribution over images; and increasing inter-subject variance of fixation distributions. The validity of these results was supported by verbal reports. This general tendency was weaker for the group of subjects who rated the image set as interesting as compared to the other group. Additionally, effects were partly mediated by subjects' motivational disposition. Finally, we found a generally strong impact of image type on eye movement parameters. We conclude that motivational tendencies linked to personality as well as individual preferences significantly affected viewing behaviour. Hence, it is important and fruitful to consider inter-individual differences on the level of motivation and personality traits within investigations of attention processes. We demonstrate that future studies on memory's impact on overt attention have to deal appropriately with several aspects that had been out of the research focus until now.

## Introduction

Overt attention under natural conditions is one current topic of human attention research [1], [2], [3], [4], [5]. Therefore, most studies implement the usage of complex and natural visual scenes to collect ecologically valid eye movement data. Given the complexity of natural stimuli, two major simplifications are commonly made:

(1) Most studies on humans' attention comprise non-repeating stimuli to exclude memory effects. Commonly, visual stimuli are presented in a nonrecurring fashion. Repeated presentation of similar or even identical stimuli is excluded in most studies to prevent possible memory effects of previous presentations. However, some studies addressed the impact of memory on overt attention: They either used simple stimulus arrays (e.g., numbers and letters) as in the contextual cueing paradigm [6] or focused on change perception in the context of classical change blindness examinations [7]. Other studies instead investigated the effect of stimulus manipulations on overt attention, such as Harding and Bloj [8], who analyzed how controlled changes to image properties affect eye gaze for repeated viewings of images. Brockmole and Henderson [9] focused on changes in eye movements when photographs were presented as mirror images after repeated presentations. To conclude, several studies utilized repeated presentation of visual stimuli in the context of scene perception and object recognition. However, to our knowledge, those studies add task or stimulus manipulations to the repeated presentation paradigm [1]. Hence, mere repeated exposure to stimuli was either assumed not to play a significant role in eye movement guidance or was not explicitly controlled. The present study addressed this fundamental issue and investigated potential changes in viewing behavior when identical natural stimuli are observed repeatedly.

(2) As a second simplification, investigations on overt attention commonly focus on universal processes [10], [11], [12], [13], [14], [15], [16], [17], [18] but neglect inter-individual differences in viewing behavior. Of course, several studies have already explored the impact of specific individual factors on eye movements, but such approaches mostly refer to sex (e.g., Mueller et al., 2008), age differences [19], [20], clinical aspects of personality such as anxiety [21], or depressive disorders [22]. In the present study, we focused on subjects' global interest in the actual activity (observing scenes repeatedly) as a motivational component that is predestined to influence memory's impact on overt attention. We therefore measured the degree to which participants perceived images as interesting. Furthermore, we also considered subjects' individual degree to maintain the actual activity. For that purpose, we selected the non-clinical personality trait ‘action orientation regarding the performance of activities’ (AOP [23]). AOP assesses the ability to stay within *interesting* activities without shifting prematurely to alternative activities. The construct AOP and corresponding items of the questionnaire closely focus on the concept of interestingness. We considered both subjects' motivational disposition and the interestingness of the stimulus material because a certain motivational (eye movement) behavior is always the result of an interplay between personal dispositions and current situational conditions [24].

To sum up, for the present eye-tracking study, we followed a complementary approach to traditional eye-tracking studies by examining the interaction between individual motivation and free viewing behaviour in a repeated presentation paradigm. In order to generalize our results to different image categories, we used natural scenes and artificial complex stimuli that cover a wide range of image types. Additionally, pink-noise images were included because these images provide the opportunity to investigate the influence of low-level image features on attention in the absense of higher order correlations in the stimuli [12].

Viewing behaviour was expected to change across repeated presentations from an initially global focus of attention to a successively local focus to scrutinize regions of interest. Individual motivation was assumed to mediate this effect of memory. The amount to which persons are motivated to further explore familiar images was expected to depend on whether individual interest in images was high or low and whether persons had the ability to stay within the actual activity or rather showed the tendency to shift prematurely to alternative activities. Consequently, we claim that individual differences on the level of motivation correlate with a person's eye movement behaviour under free viewing conditions. We hypothesize in detail as follows:

### H1: The impact of repeated presentation on viewing behavior

Repeated presentations of stimuli induce a memory trace that influences eye movement behavior, resulting in a successive locally-oriented focus of attention. This should be expressed by a result pattern of continuously increasing fixation durations as well as a decrease of saccade frequency and saccade length. The more focused viewing behavior additionally leads to a decrease in individual spread of fixation distribution. Finally, an increase of inter-subject variance of fixation distributions is expected as subjects individually select image regions for detailed analysis. Furthermore, these changes in saccadic parameters should be paralleled by subjects' introspective switch from stimulus-driven exploratory behaviour to an internal guidance of eye movements.

### H2: The impact of image type on viewing behavior

Image type has a significant effect on viewing behavior and also interacts with repeated presentation. Pink-noise images especially, being free of semantic content, are expected to elicit an overall less explorative scanning pattern (defined in (H1)) as well as a high inter-subject variance of fixation distributions.

### H3: The impact of images' interestingness on viewing behavior

Persons rating the image set as interesting should show an above average spread of eye movement behavior because they are expected to show higher motivation to extensively explore visual scenes. Higher interest in images is expected to push subjects toward more visual input of an entire visual scene and hence lead to a more extensive scanning pattern. Inter-subject variance of fixation distribution should be low if interest in images is high because image content should play an important role in eye movement guidance if images are perceived as interesting. Furthermore, fixation duration should be shorter if images were rated as interesting; saccade frequency should be higher, and saccade length should be longer. We analyze in an exploratory fashion whether this general effect of interest depends on image type.

### H4: The impact of motivational disposition on viewing behavior

The personality trait AOP as a general motivational disposition is expected to significantly moderate the effect of individual interest in images on viewing behavior. [25] already showed that state-oriented individuals do not significantly discriminate between situations of action and inaction in contrast to persons with action orientation, so we expect that the individual evaluation of whether the image set is interesting correlates with a more distinct viewing behavior within the group of action-oriented persons. Thus, action-oriented subjects who state high interest in images are expected to show the most explorative viewing behavior and the lowest inter-subject variance of fixation distributions. Moreover, differences between action and state oriented subjects should not become noticeable until an activity has been going on for some time by definition of AOP. Moreover, we assume that the interaction between subjects' motivational disposition and their interest in images also depends on image type. Pink-noise images, being free of semantic content, are expected to be most sensitive for motivation's impact on viewing behavior.

## Methods

### Participants

Forty-five university students (12 male) who were naïve to the purpose of the study participated. The average age was 24.2 years (18–48; SD = 6.68). All volunteers had normal or corrected-to-normal visual acuity and had no red-green or other colour deficiencies.

### Ethics

The study conformed to the Code of Ethics of the American Psychological Association, to the Declaration of Helsinki, and to national guidelines. Written informed consent was obtained from all participants. The study was approved by the ethics committee of the University Osnabrück.

### Stimuli

**Figure 1:**
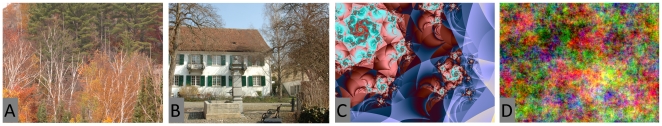
Examples of images: (a) nature; (b) urban; (c) fractal; (d) pink-noise.

The images were chosen from four categories. The first category (*nature*) contained twelve images from of the McGill Calibrated Colour Image Database [26] depicting natural environments such as open landscapes, forests, and flowers, with an absence of any man-made object (Fig. 1A). The *urban* category consisted of twelve images, such as house exteriors, streets, and vehicles. These pictures were taken with a high-resolution camera (Nikon D2X) at public places in Switzerland and were unfamiliar to participants. Scenes of both categories were free of people or writing (Fig. 1B). The third category (*fractal*) consisted of twelve software-generated fractal pictures taken from the online fractal database, chaotic n-space network (http://www.cnspace.net/html/fractals.html). All images from these three categories were scaled down or cropped to a resolution of 1280×960 pixels (4:3) and converted to bitmap format (Fig. 1C). The fourth category contained twelve pink-noise images produced as described above [12], [27]. All original images of the above described categories (nature, urban, fractal) served as base images. In the first step, they were Fourier transformed (each colour plane separately). Then the power spectrum over all images was averaged, and phase values were substituted by random values. Finally, average power spectrum and modified phase spectrum were combined by means of inverse Fourier transform. As a result, this procedure preserved the second order statistics and produced random higher order statistics. This made objects and similar assemblies undetectable in the pink-noise images (Fig. 1D).

### Apparatuses

Stimuli were presented on a 21-inch Samsung SyncMaster 1100 DF 2004 CRT Monitor (Samsung Electronics, Seoul, South Korea) in a darkened room. The display resolution was chosen to fit the image resolution of 1280×960 pixels, and the refresh rate was 85 Hz. The distance to the screen was set at 80 cm without headrest to facilitate normal viewing behaviour. The computer running the experiment was connected to the host computer (Pentium 4, Dell Inc., Round Rock, TX, USA) with the EyeLink software via a local network.

The Eye-Link II system (SR Research, Ontario, Canada) was used to record participants' eye movements. It uses infrared pupil tracking at a sampling rate of 500 Hz and compensates for head movements. Spatial resolution is <0.01°. To calibrate, participants made saccades to a grid of 13 fixation spots on the screen, which appeared one by one in a random order. The size of the point was about 0.5° of the visual angle, and the size of the 13-point grid was 25°×18°. Tracking of the eye, giving the lower validation error, started as soon as this value was below 0.35°. After each stimulus presentation, a fixation spot appeared in the middle of the screen to control for slow drifts in measured eye movements. In cases of an error being larger than 1°, calibration and validation were repeated.

Fixation locations and times were calculated online by the eye tracker system. Saccade detection was based on the following three default measures: amplitude of at least 0.1°, with a velocity of at least 30°/s and an acceleration of at least 8000°/s^2^. After saccade onset, minimal saccade velocity was 25°/s. These values had to be sustained for at least 4 min. Fixations were defined as periods without saccades [10], and the first fixation of each trial was excluded from analysis because its localization was determined by the preceding fixation spot used for drift correction.

### Experimental procedure

Participants first had to pass the Ishihara Test for Color Blindness. Then they filled out an independent subscale of the German version of the action control scale (ACS-90; German version: HAKEMP-90; [23]) and were categorized as action or state oriented. This subscale contains items assessing the ability to stay within interesting activities without shifting prematurely to alternative activities and hence is a performance related subscale (abbr.: AOP) of the ACS-90. This is a sample item from the AOP: “When I'm reading something *interesting*, I sometimes busy myself with other things for a change” (state-oriented answer). “I often stick with it for a long time” (action-oriented pole). Participants completed the AOP a second time two weeks after the experiment. Retest values correlated to a high degree with the first measurement (r = 0.89), confirming reliability of this measure of a personality trait.

Five blocks of 48 images were presented to each participant while eye movements were recorded. Each block contained the same twelve images per category presented in a pseudo-randomized manner. Presentation duration of each image was 6 s, to allow comparisons with previous studies showing complex visual scenes [1], [5], [10], [28]. Participants' instruction was to “observe the images as you want” in order to elicit an free-viewing observation mode [29] in which the viewing behavior is maximally self-determined and hence depends on the interestingness of images as well as subjects' motivational tendency (state versus action orientation). A short 5 minute break after the third presentation block when the eye tracker was removed from participants, maintained participants' alertness and avoided potential fatigue. After the break, tracking restarted with calibration and validation.

Because the items of the AOP scale focus on activities being interesting, participants subsequently saw all 48 images once more in a random order (a sixth time, now without eye-tracking) and rated the degree of interestingness of each image on a 5 point scale (1 = very not interesting to 5 = very interesting). Based on rating data, we build the factor “rating group” by median-splitting participants' overall mean interestingness rating (averaged across all images and categories). This factor divided the sample into one group, finding the set of images more interesting (RG-high; n = 24) than the second group (RG-low; n = 21).

Although Smilek et al. [4] convincingly showed that attention research can benefit from measuring a participant's subjective reports about experiences and impressions, this approach is highly unattended to date. We measured participants' impressions of the images via introspective reports to get a capacious overview of the impact of memory and motivation on overt attention. Hence, participants finally were asked non-suggestive questions: “What kind of impressions did you have during the repeated presentations of images?” Reports of their subjective impressions were recorded in written form. At the end, the participants were debriefed and informed about the purpose and details of the study.

### Independent variables

In the scope of our hypotheses, we oriented to a four factor model regarding all eye movement parameters. The first factor was the “presentation” run (5 levels); the second factor was the “image category” (4); the third factor was the personality trait “AOP” (2); and the “rating group” (2) was the fourth factor.

### Dependent variables

Eye-tracking data first were analyzed regarding fixation duration, saccade frequency, and saccade length. Second, individual distribution of fixations over images was investigated using corrected entropy-measure quantifying spread. Finally, we analyzed inter-subject fixation distributions that indicate inter-subject reliability of fixations. For all subsequent analysis, the first fixation was excluded as it is solely due to the preceding fixation spot used for drift correction.

### Saccade parameters

For fixation duration, all fixations made on an image were taken into account, excluding fixations whose duration differed by more than two standard deviations from the grand mean. This limits the potential influence of outliers. Fixation duration was calculated by the eye tracking system online. Saccade length was operationalized by Euclidean distance between two consecutive fixations marked by their coordinates in the two-dimensional image space. Saccade frequency is the number of all valid saccades per unit time. If multiple tests were necessary to check specific effects of interest, the alpha-level was Bonferroni-adjusted.

### Individual fixation distributions

To investigate the spread of fixation distribution independent of specific geometrical arrangements, we employed the concept of entropy. The fixation distribution map of subject *s* viewing image *i* in presentation run *p* was convolved with a Gaussian kernel. The full width at half maximum (FWHM) of the Gaussian kernel defining the size of the patch was set to 1° of visual angle. Then the entropy *E* of the resulted fixation density map (FDM) was calculated with standard MATLAB (MathWorks, Inc.) function according to



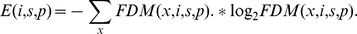
Higher values indicate a more spread out distribution. Extreme values occur for singular distributions (minimum) and a flat distribution (maximum). These values were averaged over images (of a category) or subjects (personality traits) as appropriate.

However, estimators of entropy are influenced by sample size [30], [31]. Especially in cases of few fixations, measures of entropy are biased, and without correction, return to low estimates [32]. As no general unbiased estimator is available, we equalized the bias using a bootstrapping technique: For each trial with more than 9 fixations, we randomly sampled 9 fixation points with 100 repetitions and calculated the corresponding entropy value *E* each time. Finally, the mean entropy 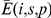 for each trial was computed. The sample size was set to nine as a too small target number for downsampling would lead to a too large loss of power. Trials with a fixation number lower than 9 (5.5%) were excluded from bootstrapping analysis. This procedure results in a valid comparison of entropy measures of different sample sizes. Importantly, absolute entropy values are not relevant as they depend on image resolution as well as on the size of the Gaussian kernel used for convolution.

### Inter-subject variance of fixation distributions

To quantify the reliability of inter-subject fixation distributions with respect to image *i*, we calculated the inter-subject variance of fixation density maps *V(i)* by
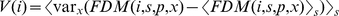
It calculates how much fixation behavior of subjects deviate from average fixation behavior of subjects on specific image *i.* The higher *V(i)*, the more variance between individual fixation distributions exist and the lower the inter-subject reliability of fixation distributions. *V(i)* is robust toward the central fixation bias.

### Subjective impressions

The subjects' statements were analyzed via a three-step qualitative data analysis procedure [33]. This procedure includes first an explication of statements. Second, statements were categorized by two independent raters with the aid of a given category-system. Finally, a frequency analysis was computed. Inter-rater-reliability was high (Cohen's Kappa = .93), and in the few cases of absent agreement, a consensual categorization was required to allow frequency analysis. The six categories consisted of statements expressing (1) participants' impression that salient regions or objects seemed to attract attention automatically even during repeated presentation, (2) strategy to obtain an overview about image content during first presentation(s), (3) strategy to focus on certain image regions of interest during later presentations, (4) the impression that the experimental course of repeated presentations induced boredom, (5) the impression that a certain image category was boring, and finally (6) a category containing all remaining statements such as feelings or sentence fragments.

## Results

First, we checked whether splitting the stimulus sample regarding their interestingness rating was valid independent of the subject group. For that purpose, ratings were averaged across all images of one category, and then a 4×2 (image category x AOP) repeated measures ANOVA (Greenhouse-Geisser applied) was calculated. AOP was introduced to exclude potential confounds between image ratings and this personal factor. We only obtained a main effect of image category [F(2.697, 110.566) = 23.438; p<.001] with a significantly lower interestingness rating for pink-noise images (p<.001; urban: M = 3.06; *nature*: M = 2.94; *fractal*: M = 2.81; *pink-noise*: M = 1.71). Because no rating differences between action oriented (AOP-action; n = 17) and state oriented participants (AOP-state; n = 28) as well as no interaction were found, this legitimised the factor of a “rating group” of images.

For all further parametric tests based on the assumption that the data follow a normal distribution, Kolmogorov-Smirnov tests were introductorily calculated. This was done for all cells of the 5×4×2×2 design [presentation x image category x AOP x rating group] and all dependent variables separately before corresponding 5×4×2×2 repeated measurement ANOVAs (Greenhouse-Geisser applied) were calculated. No violation of this assumption was found (all p>.24), and parametrical tests were thus appropriate. In the case of significant main effects, a post-hoc Bonferroni-adjusted t-test calculated for pairwise comparisons of factor levels. Results are depicted in the corresponding figures. Effect sizes as indicators for practical significance are reported by means of partial eta squared (η_p_^2^).

### The impact of repeated presentation on viewing behavior (H1)

**Figure 2:**
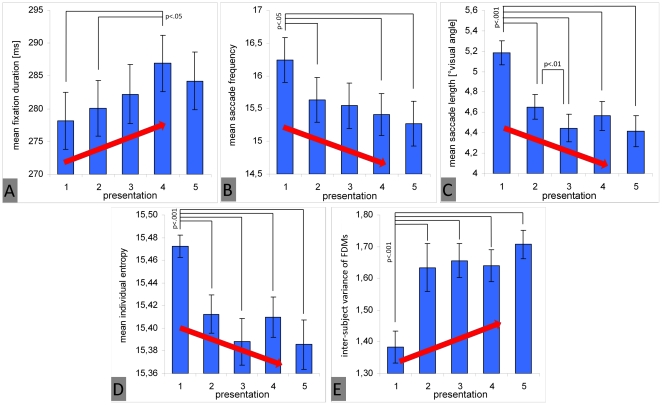
Main effect of repeated image presentation on eye movement parameters. The figure shows fixation duration (A), saccade frequency (B), saccade length (C), individual fixation distribution (individual entropy, D), and inter-subject variance of fixation density maps (E). Red arrows indicate changes in parameters predicted by hypothesis 1. Vertical lines on top of bars indicate standard error of the mean. Significant differences between presentation runs (post-hoc Bonferroni adjusted t-tests) are marked.

With respect to fixation duration and inter-subject variance of fixation density maps, we expected an increase across repeated presentation of images. With respect to saccade frequency, saccade length, and individual fixation distribution (entropy), we expected a decrease over time. The ANOVA for fixation duration revealed a significant increase across repeated presentations and a maximum at a fourth presentation [F(2.54, 103.96) = 3.89; p<.05; η_p_^2^ = .09] (fig. 2A). In contrast, saccade frequency showed a significant decrease across repeated presentation [F(2.44, 99.98) = 6.61; p<.001; η_p_^2^ = .14] (fig. 2B) as well as saccade length [F(1.921, 78.76) = 28.43; p<.001; η_p_^2^ = .41] (fig. 2C). With respect to the mean individual spread of fixation distribution (entropy), we found a significant reduction from the first presentation to all later presentations [F(2.38, 97,60) = 15.08; p<.001; η_p_^2^ = .27], and subjects' viewing behavior was more explorative during the initial presentation (fig. 2D). Finally, the inter-subject variance of fixation density maps significantly increased from first to second presentation and remained comparably high during the later presentations [F(3.43, 301.95) = 26.11; p<.001; η_p_^2^ = .23] (fig. 2E).

**Figure 3:**
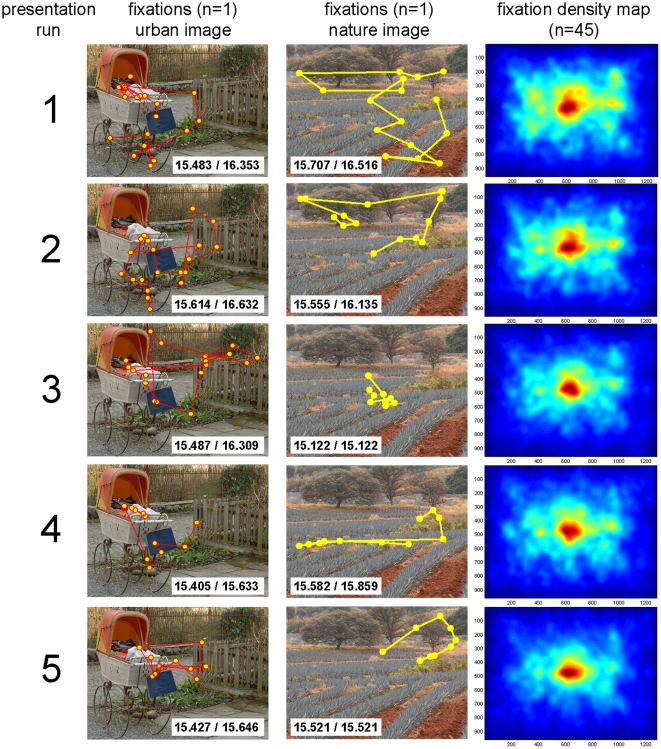
Examples of fixation distribution maps for single subjects on selected urban and nature images as well as total fixation distribution maps for all five presentation runs. The spread of fixation distributions quantified by means of entropy values is depicted (left entropy value  =  corrected via bootstrapping/right entropy value  =  exact value without correction for number of fixations). The right column depicts fixation distribution maps, including all fixations of the sample to illustrate a stronger central bias at later presentation runs.

In summary, the presented results confirm the predictions of hypothesis 1. Repeated presentations of complex scenes induce a memory trace that is responsible for a general, successive, locally-oriented focus of attention. Therefore, the biggest change occurred between initial and second observation and is expressed by a result pattern of increasing fixation durations, a decrease of saccade frequency and saccade length, and a reduced individual fixation distribution. Moreover, inter-subject variance of fixation distribution increased with further presentation runs and confirms the assumption that subjects differ regarding scene regions they intended to explore further. Figure 3 depicts examples of raw eye movement data to illustrate changes across repeated presentations.

### Subjective impressions

Overall, 161 statements were recorded from 45 participants. Statement distribution over categories revealed that 38 of 45 participants provided at least 1 statement falling into category 3, which contains statements that express participants' strategy to focus on certain image regions of interest during later presentations. A further 30 statements fell into the refuse category 6; 20 statements into category 4 (experimental course of repeated presentations induced boredom); and 14 into category 5 (a specific image category bored). Eight statements belong to categories 1 (salient regions or objects seemed to attract attention automatically during repeated presentation) and 2 (strategy to get an overview about image content during first presentation), respectively. A comparison between participants of different motivational state measured by interestingness rating of images and by AOP did not reveal considerable differences in statement frequencies. Frequencies were similar in all categories [χ^2^ = 12.40; p = .64]. Hence, according to hypothesis 1, systematic changes in saccadic parameters were parallel to an introspective switch from stimulus-driven exploratory behaviour to an internal guidance of eye movements.

### The impact of image type on viewing behavior (H2)

We expected a less explorative scanning pattern on pink noise images expressed by longest fixation duration, lowest saccade frequency, shortest saccade length, and lowest individual entropy. Moreover, inter-subject variance of fixation distributions was expected to be maximal due to the absent of any semantic content in pink-noise images.

**Figure 4:**
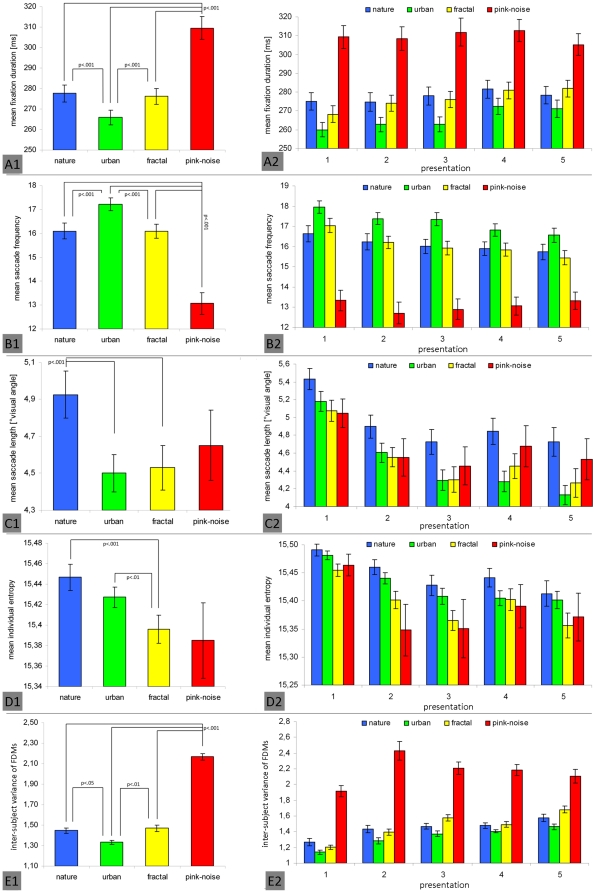
Significant main effect of image type on eye movement parameters (left column) and its significant interaction with repeated image presentation (right column). Viewing behavior was measured by means of fixation duration (A), saccade frequency (B), saccade length (C), individual fixation distribution (entropy, D), and inter-subject variance of fixation density maps (E). Vertical lines on top of bars indicate standard error of the mean. Significant differences between presentation runs (post-hoc Bonferroni adjusted t-tests) are marked.

With respect to fixation duration, the main effect of image category was found [F(1.90, 78.04) = 111.66; p<.001; η_p_^2^ = .71] with shortest fixation durations on urban scenes and longest durations on pink-noise images (fig. 4-A1). Differences are significant between all four image categories (all p<.001), except nature and fractal images. A main effect was also found for saccade frequency [F(1.70, 69.54) = 142.17; p<.001; η_p_^2^ = .78]. All image categories except nature and fractal images differed significantly (all p<.001). The highest fixation frequency was found at urban scenes and lowest at pink-noise images, confirming our prediction (fig. 4-B1). Saccade length also differed significantly between image types [F(1.43, 58.77] = 6.38; p<.01; η_p_^2^ = .14] and was significantly longer on nature images than on urban and fractal images (both p<.001; fig. 4-C1). However, and in contrast to the prediction, pink-noise images did not elicit the shortest saccades. With respect to individual fixation distribution, we also found a significant main effect [F(1.17, 47.80) = 4.58; p<.05; η_p_^2^ = .10] (fig. 4-D1). Entropy was significantly higher on nature images than on urban images (p<.001) and fractal images (p<.01). Although mean entropy was minimal on pink-noise images as predicted, no significant differences to any other image category were revealed due to the huge intra-category variance of entropy values. Finally, inter-subject variance of fixation distributions differed significantly between all image categories, except urban nature and fractal images [F(3, 44) = 194.60; p<.001; η_p_^2^ = .93] with highest inter-subject variance on pink-noise images (fig. 4-E1).

These results clearly confirm the expected impact of image type on free viewing behavior. As formulated in hypothesis 2, the most locally-oriented focus of attention was elicited by pink-noise images paralleled by maximal variance between subjects regarding all dependent variables. Moreover, the inter-subject variance of fixation distribution was maximal on pink-noise images, indicating that the absence of semantic content leads to a notably individual scanning pattern. Moreover, effects of image type were significantly moderated by repeated presentation with respect to all parameters as depicted in figure 4 (A2–AE) (all p<.05; all η_p_^2^>.050).

### The impact of interest on viewing behavior (H3)

We expected that participants who rated the image set as interesting, in contrast to those who did not, would show a more globally-oriented eye movement behavior expressed by shorter fixation durations, higher saccade frequencies, longer saccades, higher entropy values, and a lower inter-subject variance of fixation distributions.

**Figure 5:**
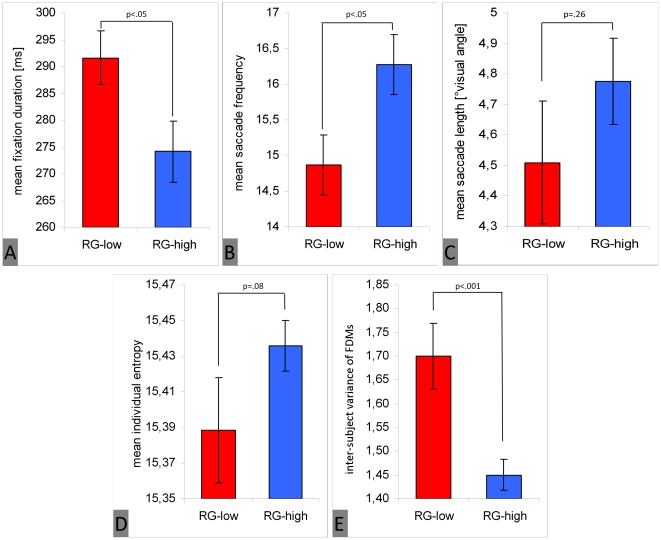
Main effect of subjects' global interest in the image set on eye movement parameters. The figure shows fixation duration (A), saccade frequency (B), saccade length (C), individual entropy (D), and inter-subject variance (reliability of fixation density maps, E). Blue bars represent participants who rated the image set as interesting (RG-high), and red bars represent participants who rated images as not interesting (RG-low). Vertical lines on top of bars indicate standard error of the mean.

We found that subjects who rated the image set as interesting showed significantly shorter fixation durations [F(1, 41) = 7.07; p<.05; η_p_^2^ = .15 ] (fig. 5A), higher saccade frequencies [F(1, 41) = 6.73; p<.05; η_p_^2^ = .14] (fig. 5B), and mean saccade lengths longer only by trend [F(1, 41) = 1.33; p = .26; η_p_^2^ = .03] (fig. 5C). The difference between both groups regarding individual entropy was slightly significant [F(1, 41) = 3.17; p = .08; η_p_^2^ = .07], with a higher spread of fixation distributions for subjects who rated images as interesting (fig. 5D). Finally, inter-subject variance of fixation distribution was significantly lower in this group as predicted [F(1, 88) = 126.91; p<.001; η_p_^2^ = .59]. The inter-subject reliability of fixation distributions was higher (fig. 5E).

These results confirm the prediction derived from hypothesis 3. We found a more globally- oriented focus of attention (shorter fixation durations, higher saccade frequencies, and more extensive fixation distributions) for those participants interested in the image set. However, when interest in images was high, inter-subject variance of fixation distribution was low. This indicates that in the case of high internal motivation (interest), viewing behavior is guided strongly by the external image content.

Moreover, we expected that the difference in viewing behavior between subjects interested in images and those who were not should interact with image type. Persons who rated images as interesting were expected to show an above average spread of eye movement behavior, shorter fixation durations, longer saccade lengths, higher saccade frequencies, and a lower inter-subject variance of fixation distributions than subjects who rated images as not interesting. But these effects should be moderated by image type with maximal effects on pink-noise images.

**Figure 6:**
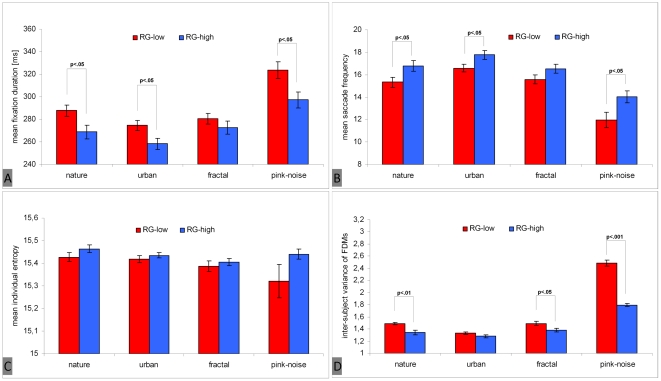
The effect of subjects' global interest in the image set on eye movement parameters depending on image type. Significant interactions were found regarding fixation duration (A), saccade frequency (B), individual entropy (C), and inter-subject variance (reliability of fixation density maps, D). Blue bars represent participants who rated the image set as interesting (RG-high), and red bars represent participants who rated images as not interesting (RG-low). Vertical lines on top of bars indicate standard error of the mean.

ANOVAs revealed significant interactions as predicted between “image category” and “rating group” with respect to fixation duration [F(1.90, 78.04) = 6.17; p<.01; η_p_^2^ = .13] (fig. 6A), saccade frequency [F(1.696, 69.542) = 4.85; p<.05; η_p_^2^ = .11] (fig 6B), individual fixation distribution quantified by entropy [F(1.17, 47.80) = 4.33; p<.05; η_p_^2^ = .10] (fig. 6C), and inter-subject variance of fixation distributions [F(3, 88) = 44.53; p<.001; η_p_^2^ = .60] (fig. 6D).

### The impact of motivational disposition on viewing behavior (H4)

We also expected that the trait AOP in combination with the repeated presentation of stimuli would influence the effect of interest in images. The individual evaluation of whether the images are interesting should correlate with a more distinct viewing behavior between RG-low and RG-high within the group of action-oriented subjects. Additionally, this difference between action and state oriented subjects should be noticeable only after an activity has gone on for some time. Therefore, action-oriented subjects who have high interest in images are expected to show the most explorative viewing behavior and the lowest inter-rater variance of fixation distributions.

**Figure 7:**
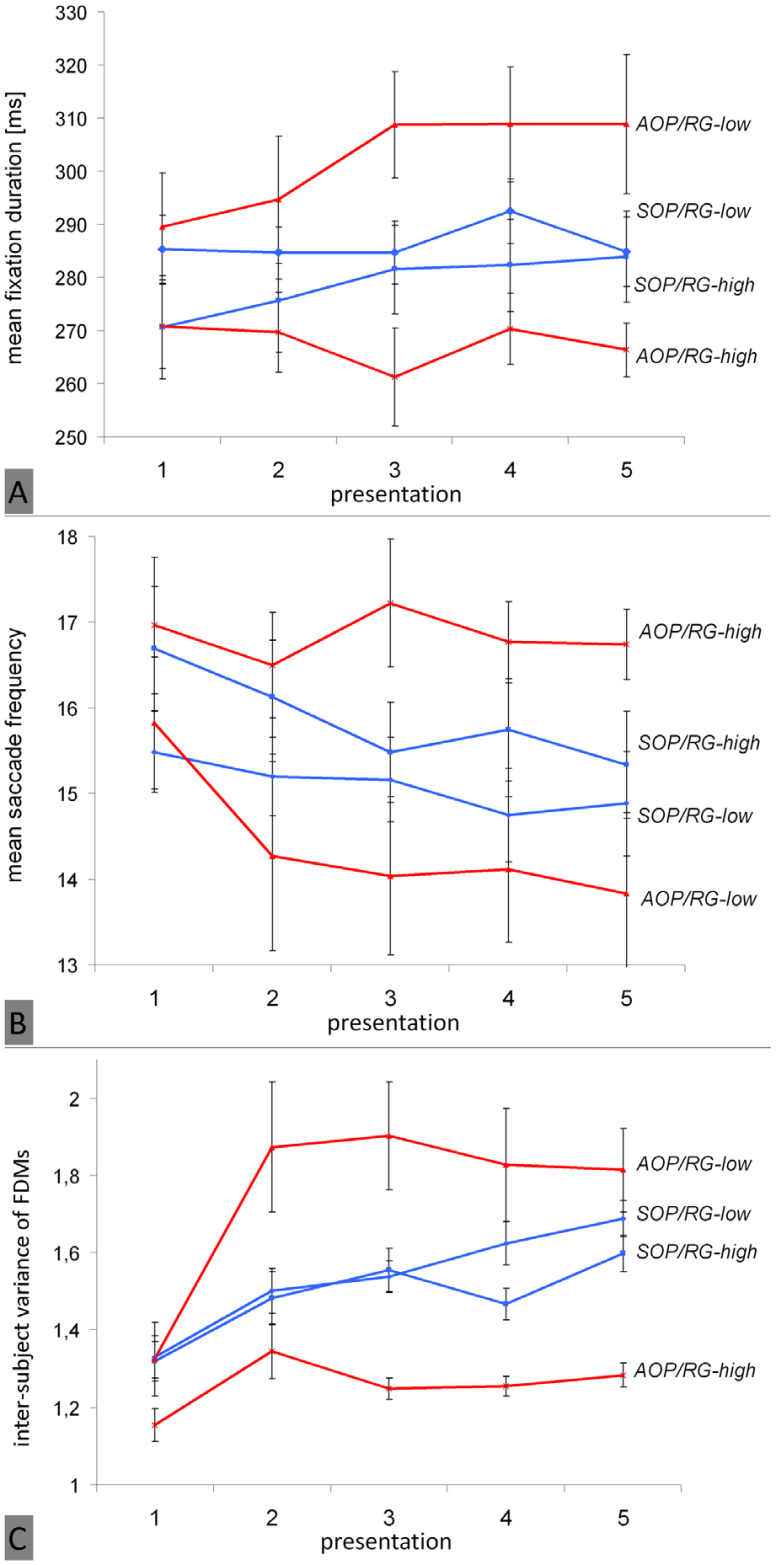
Significant three-way interaction between repeated image presentation, subjects' global interest in the image set and subjects' motivational disposition (AOP; action orientation during performance of activities) regarding several eye movement parameters. The figure shows fixation duration (A), saccade frequency (B), and inter-subject variance of fixation density maps (C). Figures describe course of eye movement changes with respect to four different motivation groups: Red lines represent mean values of action-oriented participants (AOP), and blue lines indicate state-oriented participants (SOP). Both groups are additionally subdivided into participants who rated images as interesting (RG-high) and those who did not (RG-low). Vertical lines indicate standard error of the mean.

To test this hypothesis, the potential three-way interactions of the “rating group,” “AOP,” and “presentation” were analyzed for all dependent variables. With respect to fixation duration [F(2.54, 103.96) = 5.05; p<.01; η_p_^2^ = .11] as well as to saccade frequency [F(2.44, 99.98) = 3.30; p<.05; η_p_^2^ = .07], significant three-way interactions were found. As depicted in figure 7A, participants who rated interestingness of images low (RG-low) had longer fixation durations at all presentation runs than participants of the other group (RG-high). Remarkably, these differences were highly influenced by the personality trait “AOP.” Differences in fixation duration were greater for action- oriented subjects (AOP) than for state-oriented ones (SOP). Furthermore, this interaction is clearly moderated by “presentation,” and the effect of AOP increased with repeated presentations. Action- oriented participants who rated images as interesting showed the shortest fixation durations in all blocks. Additionally, higher fixation durations in RG-low coincide with lower fixation frequencies whereas differences became larger only for action-oriented subjects at later presentation runs (see fig. 7B). However, no differences between groups were found with respect to saccade length [F(1.92, 78.76) = 0.43; p = .64; η_p_^2^ = .01] and individual entropy [F(2.38, 97.60) = 0.67; p = .54; η_p_^2^ = .02]. Finally, inter-subject variance of fixation distributions showed the predicted significant three-way interaction between “presentation,” “AOP,” and “rating group” [F(10.57, 620.01) = 4.10; p<.001; η_p_^2^ = .07] (fig. 7C). Participants of RG-high showed lower inter-subject variance of fixation density maps than subjects of RG-low at all presentation runs, but differences between RG-high and RG-low are larger within the group of action-oriented subjects.

**Figure 8:**
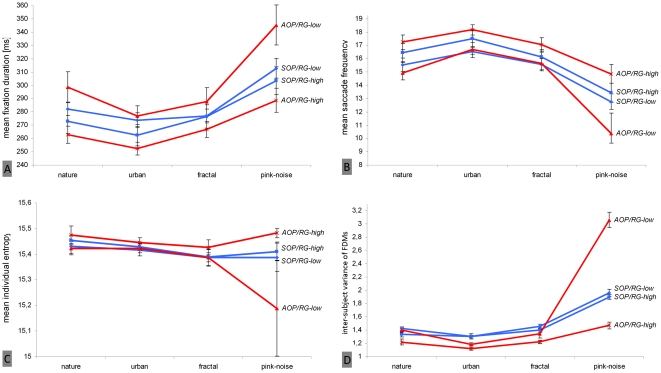
Significant three-way interactions between image type, subjects' global interest in the image set, and subjects' motivational disposition (AOP; action orientation during performance of activities) regarding several eye movement parameters. Figures describe the course of significant eye movement changes with respect to four different motivation groups on the level of fixation duration (A), saccade frequency (B), individual spread of fixation distribution (entropy, C), and inter-subject variance of fixation density maps (D). Red lines represent mean values of action-oriented participants (AOP), and blue lines indicate state- oriented participants (SOP). Both groups are additionally subdivided into participants interested in images (RG-high) and those who were not (RG-low). Vertical lines indicate standard error of the mean.

Finally, we analyzed whether the large differences in viewing behavior on pink-noise images found between persons rating images as interesting and those who did not (see above; fig.6) were additionally larger within the group of action-oriented subjects. In fact, three-way interactions between “image category,” “AOP,” and “rating group” were obtained for fixation duration [F(1.90, 78.04) = 3.66; p<.05; η_p_^2^ = .08] (fig. 8A), saccade frequency [F(1.696, 69,542) = 5.648; p<.01; η_p_^2^ = .12] (fig. 8B), individual fixation distribution [F(1.17, 47.80) = 3.66; p = .06; η_p_^2^ = .08] (fig. 8C), and inter-subject variance of fixation distributions [F(9, 176) = 49.76; p<.001; η_p_^2^ = .72] (fig. 8D). Differences on pink-noise images were obviously larger between rating groups for action-oriented subjects.

Consequently, even on the level of image categories, we found evidence of an effect of individual motivational disposition on viewing behavior. In the first instance, pink noise images free of semantic content seemed eligible to evoke discriminative visual scanning between persons with different motivational dispositions and interests in actual activity. Action-oriented subjects who rated images as interesting showed the most explorative viewing behavior and simultaneously produced the smallest inter-subject variance of fixation distribution independent of image type.

Because pink-noise images were sensitive to individual motivation, we finally checked whether the above reported main effects of repeated presentation (hypothesis 1) and “rating group” (hypothesis 3) as well as the three-way-interaction between “rating group,” “AOP,” and “presentation” (hypothesis 4) would be still present after rejection of pink-noise images. In fact, all reported effects remained significant (all p<.05), and hence those effects are not present only due to the specific category of pink-noise images.

### Validity check of main effects

**Figure 9:**
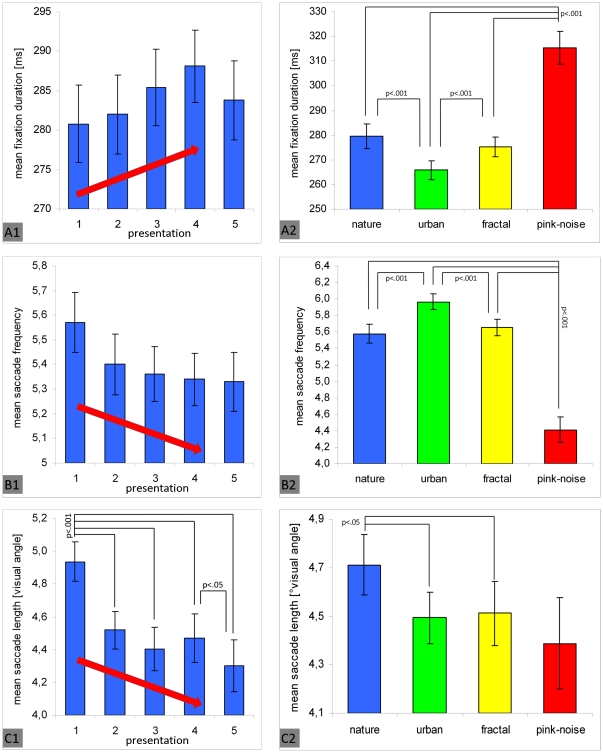
The effects of repeated image presentation (left column) and image type (right column) on eye movement parameters for the first two seconds of image observation. The result pattern found regarding fixation duration (A), saccade frequency (B), and saccade length (C) are exact replications of the original effects found for the whole observation interval of six seconds. Vertical lines on top of bars indicate standard error of the mean. Significant differences between presentation runs (post-hoc Bonferroni adjusted t-tests) are marked.

**Figure 10:**
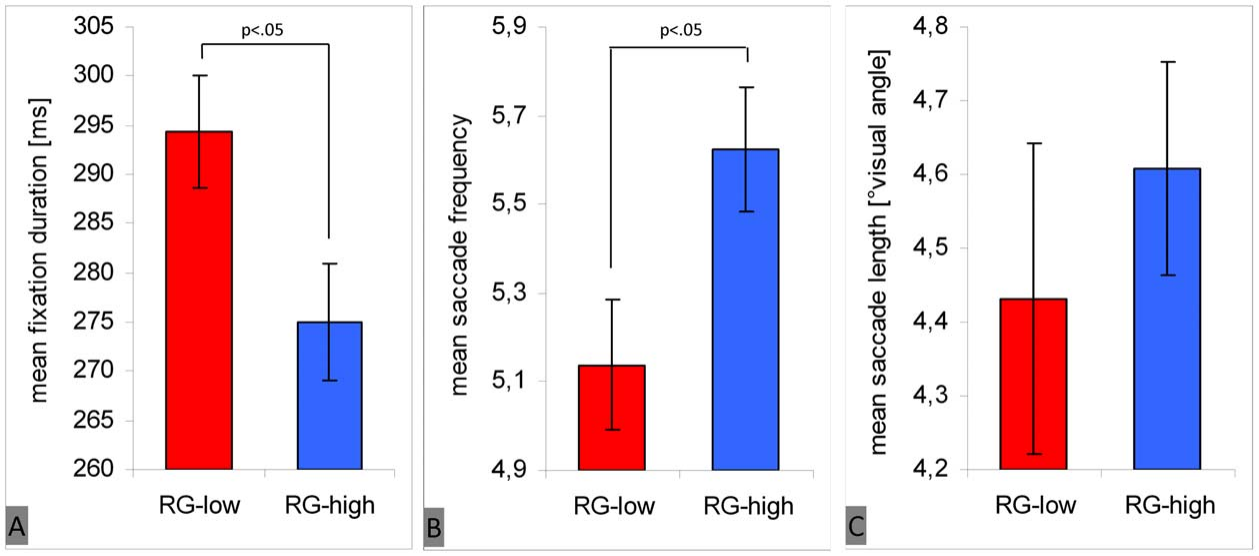
The effect of subjects' global interest in the image set on eye movement parameters for the first two seconds of image observation. The result pattern found regarding fixation duration (A), saccade frequency (B), and saccade length (C) are exact replications of the original results found for the whole observation interval of six seconds. Vertical lines on top of bars indicate standard error of the mean.

We found specific main effects of repeated presentation, image type, and subjects' interest in the image set on viewing behavior. To check whether these results are stable and not only derived from the fixed observation interval of six seconds, we additionally limited corresponding analysis to the first two seconds of image observation. Due to the smaller number of fixations involved, the statistical power is reduced, and entropy as well as inter-subject variance of fixation density maps cannot be analyzed appropriately. Nevertheless, as depicted by figures 9 and 10, no qualitative change regarding fixation duration, saccade frequency, and saccade length was observed. The main effects of repeated presentation (fig. 9 A1–C1) and image type (fig. 9 A2–C2) were replicated exactly as well as the difference between subjects who were interested in the image set and those who were not (fig. 10 A–C). Although power was reduced, all effects actually reached significance [all p.<.05; all η_p_^2^>.08] except the effect of repeated presentation on fixation duration and the effect of subject interest in images on saccade length. Consequently, the present results seem to be highly valid independent of the fixed observation duration.

## Discussion

In the current eye-tracking study, we presented complex images of different types repeatedly to pursue changes in viewing behavior. Additionally, we considered participants' individual context by measuring their motivational disposition regarding performance of activities as well as by their interest in the image set.

We found that participant-viewing behavior changed with respect to several eye movement parameters across repeated presentations. As a general tendency, duration of fixations continuously increased, and fixation frequency as well as saccade length decreased, indicating that the focus of attention became increasingly local. Importantly, fixation duration did not decrease, and saccade frequency did not increase, although increasing familiarity of images could speed up the information process. The present results rather suggest that subjects scrutinized individual regions of interest at later presentations. Thus, changes occurred quickly and were largest between the initial and second presentation. Obviously, the step from the initial unfamiliarity of images to familiarity in the second presentation affected viewing behavior maximally. The extensive (global) scanning of the whole image found at first presentation is more proper to get an overview of the complete scene. By reducing the width of attention's focus across repeated presentations, the observer samples more detailed information about a specific image area. This interpretation is additionally supported by a decrease of single subject fixation distribution over images. Interestingly, these changes in eye movement parameters seem to be non-linear as differences between later presentations were marginal or absent. Moreover, the significant increase in inter-subject variance of fixation distributions over time indicates a more internal guidance of eye movement behavior and hence a lower inter-subject reliability of fixated image regions at later presentations. Subjects' introspection also indicated this change in attention focus: About 85% of participants explicitly noted that their scanning behavior became more locally-oriented between the first and later presentations.

We can generalize this effect of repeated presentation to different image categories. The focus of attention became increasingly local on natural and urban scenes as well as on artificial fractal and pink-noise images. Moreover, this effect of repeated presentation on viewing behavior was also present when observation duration was limited to a two-second interval. A similar result was found by Hooge and Erkelens [34], who found that shortening the presentation time of stimuli did not affect fixation duration. Hence, the effect of repeated presentation and memory traces seems stable independent of observation time.

In addition to this memory effect on viewing behavior, we found a strong impact of image type. The most locally-oriented focus of attention was elicited by pink-noise images paralleled by maximal variance between subjects regarding all dependent variables. The inter-subject variance of fixation distribution was maximal on pink-noise images, indicating that the absence of semantic content leads to a notably individual scanning pattern.

Effect sizes vary between the influential factors on viewing behavior and eye movement parameters. For example, the influence of image type on fixation duration was greater than the impact of repeated presentation. This result is compatible with the findings of Frey, Honey and König [35], who suggested that the impact of basic image features on viewing behavior highly depends on image type. However, memory's impact on saccade length was greater than the impact of image category. Changes in saccade frequency were significant but small. Overall, effect sizes are similar to effect sizes reported for pop-out tasks [34], web page observation [11], [36], or for reading tasks [37]. However, especially in the context of viewing behavior on complex scenes, further studies are necessary to provide a broader basis for effect size estimation.

Given the potential impact of motivation on viewing behavior, we hypothesized differences in viewing behavior between subjects derived from different motivational states. In detail, participants who perceived the image set as interesting (RG-high) were expected to show a more global focus of attention than subjects who rated interestingness of the stimulus material as low (RG-low). Specifically, subjects allocated to the RG-high group were expected to show higher motivation to explore further an image, as they would take more pleasure in such an exploration. Results confirm this prediction: Significant differences occurred for all analyzed eye movement parameters between RG-high and RG-low. Participants of group RG-low showed a more locally- oriented scanning pattern expressed by longer fixation duration, lower saccade frequency, and narrower fixation distributions, independent of image category. Saccade length in contrast was similar for all subjects, and consequently, the visual step size appears to be less sensitive to differences in subjects' motivational state. Moreover, inter-subject variance of fixation distributions in group RG-low was significantly higher, indicating that subjects who had a lower interest in images were less guided by image content. This led to a lower inter-subject reliability of fixated image regions. In contrast, subjects who reported high interest in images showed a lower inter-subject variance of fixation distributions.

Moreover, we also found an effect of subjects' motivational disposition on viewing behavior. We decided to measure a specific personality trait linked to motivation by means of action orientation during performance of activities (AOP). But the main construct of action orientation itself is widespread in motivational psychology [38], [39], [40], [41]. AOP assess the ability to stay within *interesting* activities without shifting prematurely to alternative activities. It is thus necessary to consider the interestingness of the actual activity to assess the influence of AOP on (viewing) behavior. No main effect of AOP was found. However, the differences in eye movement parameters between subjects interested in images and those who were not were dependent upon AOP. Within the subgroup of action-oriented subjects, the effect of the interest in images was great in contrast to the state oriented subgroup. Additionally, in the present study, action-oriented persons who did not perceive the image set as interesting are characterized by the most local focus of attention, the longest fixation durations, the lowest saccade frequencies, and the highest inter-subject variance of fixation density maps. Consequently, results showed that action-oriented persons discriminated more between interesting and non-interesting activities than did state-oriented persons. This paralleled McElroy and Kingdom [25], who, regarding the eye movement parameter, showed that state-oriented individuals did not significantly discriminate between situations of action and inaction compared to persons with action orientation.

Finally, the influence of individual motivation on the impact image type on viewing behavior provides further evidence for the postulated mediating role of motivation. Pink noise especially evoked differences in visual scanning between persons with different motivational disposition and interest in actual activity.

The present study provides evidence that actual motivation is an influential factor in eye movements and that situation-independent personality traits are potential determining factors of viewing behavior. Future research on overt attention will be beneficial if it considers motivational states of participants. Moreover, the repeated presentation approach delivers an elegant way to investigate memory-based changes in viewing behaviour by keeping stimulus properties constant; it is also ecologically more valid than single exposures to stimuli because in everyday life, we are continually confronted with repeated visual impressions.
